# Perception of Biological Motion in Schizophrenia and Healthy Individuals: A Behavioral and fMRI Study

**DOI:** 10.1371/journal.pone.0019971

**Published:** 2011-05-20

**Authors:** Jejoong Kim, Sohee Park, Randolph Blake

**Affiliations:** 1 Department of Brain and Cognitive Sciences, Seoul National University, Seoul, Korea; 2 Department of Psychology, Vanderbilt University, Nashville, Tennessee, United States of America; 3 Vanderbilt Vision Research Center, Vanderbilt University, Nashville, Tennessee, United States of America; University of Leuven, Belgium

## Abstract

**Background:**

Anomalous visual perception is a common feature of schizophrenia plausibly associated with impaired social cognition that, in turn, could affect social behavior. Past research suggests impairment in biological motion perception in schizophrenia. Behavioral and functional magnetic resonance imaging (fMRI) experiments were conducted to verify the existence of this impairment, to clarify its perceptual basis, and to identify accompanying neural concomitants of those deficits.

**Methodology/Findings:**

In Experiment 1, we measured ability to detect biological motion portrayed by point-light animations embedded within masking noise. Experiment 2 measured discrimination accuracy for pairs of point-light biological motion sequences differing in the degree of perturbation of the kinematics portrayed in those sequences. Experiment 3 measured BOLD signals using event-related fMRI during a biological motion categorization task.

Compared to healthy individuals, schizophrenia patients performed significantly worse on both the detection (Experiment 1) and discrimination (Experiment 2) tasks. Consistent with the behavioral results, the fMRI study revealed that healthy individuals exhibited strong activation to biological motion, but not to scrambled motion in the posterior portion of the superior temporal sulcus (STSp). Interestingly, strong STSp activation was also observed for scrambled or partially scrambled motion when the healthy participants perceived it as normal biological motion. On the other hand, STSp activation in schizophrenia patients was not selective to biological or scrambled motion.

**Conclusion:**

Schizophrenia is accompanied by difficulties discriminating biological from non-biological motion, and associated with those difficulties are altered patterns of neural responses within brain area STSp. The perceptual deficits exhibited by schizophrenia patients may be an exaggerated manifestation of neural events within STSp associated with perceptual errors made by healthy observers on these same tasks. The present findings fit within the context of theories of delusion involving perceptual and cognitive processes.

## Introduction

Humans are remarkably adept at perceiving the actions and intentions of others, an especially important skill befitting out highly social nature [1]. Called biological motion perception, this skill has been extensively studied in the laboratory using point-light (PL) animations of human activity portrayed exclusively by dots of light depicting the trajectories of the limbs of an actor's body [2]. Upon viewing PL animations, most people have no trouble perceiving subtle characteristics of the PL actor including the actor's gender [3], [4], identity [5], and social signals such as mood [6]. This paper deals with perception of biological motion in people with schizophrenia, a psychotic disorder characterized by debilitating deficits in a multitude of cognitive and social domains.

Psychophysical studies indicate that schizophrenia patients exhibit deficits on a variety of visual tasks including judgment of spatial location [7], discrimination of spatial frequencies [8], and detection of visual motion [9]–[12]. One particularly intriguing deficit uncovered in recent work from our laboratory was that schizophrenia patients exhibit impaired performance on a task involving discrimination of ordinary PL sequences of biological motion from sequences in which the spatial location of the dots were perturbed [13]. In this study, we used a discrimination task in which, on each test trial, patients viewed either a PL animation of a person engaged in one of several, familiar activities (e.g. walking or running) or an animation consisting of the same PL motions spatially and temporally scrambled to perturb the normal kinematics of the activity; the order of animations over trials was random and following each trial the patient categorized the animation as biological or perturbed. Signal detection analyses revealed significantly lower categorization performance (*d*') by the schizophrenia patients compared to matched control participants, and this reduction in performance arose primarily from their abnormally high false alarm rates (i.e., judging a scrambled sequence as normal biological). These results imply that patients may be generally less sensitive to the kinematics defining normal, coordinated motion of the human body. If people with schizophrenia are indeed less sensitive to the kinematics defining human social actions, this could represent a significant perceptual component related to the multiple deficits in the social domain in schizophrenia [14]. Such a social perceptual deficit could also imply the existence of abnormalities in brain structures thought to be involved in perception of biological motion [15].

Because of the potentially important implications of our initial results, we performed three experiments aimed at documenting the nature and possible neural bases of impaired perception of biological motion in schizophrenia, using refined psychophysical techniques coupled with fMRI brain imaging. Experiments 1 and 2 were designed to elucidate the perceptual bases of impaired biological motion perception in schizophrenia. Results from those two experiments, in turn, set the stage for Experiment 3. This was a brain imaging study focusing on the posterior portion of the superior temporal sulcus (STSp), a brain region widely considered to be a lynchpin in a network of areas involved in registration of socially relevant sensory information [16]–[21].

## Experiment 1: Detection of biological motion embedded in noise

In Experiment 1 we used a two-alternative, forced-choice method (2AFC) to estimate thresholds for detection of biological motion perception for PL sequences in noise dots that obscured the spatio-temporal coherence of the dozen or so PL dots describing human activity [22]–[24]. Two successive motion sequences were presented, one in each of two intervals defining a trial: one interval contained a dot sequence defining a biological activity in noise and the other contained a scrambled version of that sequence also embedded in noise, and the participants indicated which of the two intervals contained a biological sequence. By varying the number of noise dots over trials using a staircase procedure, we determined the minimum signal-to-noise-ratio supporting above chance performance on this 2AFC task requiring discrimination of PL biological motion.

### Materials and Methods

#### Ethics Statement

In this and the following two experiments, written informed consent was obtained from all participants after they were given a complete description of the study. The Institutional Review Board of Vanderbilt University approved the protocol and consent procedure.

#### Participants

Fifteen outpatients (7 females and 8 males) who met the DSM-IV [25] criteria for schizophrenia were recruited from private psychiatric facilities in Nashville, Tennessee. Exclusion criteria were head injury, neurological disorders, substance use within the past 6 months, and IQ<85. Clinical symptoms were assessed with the Brief Psychiatric Rating Scale (BPRS)[26]. Positive and negative symptoms were assessed using the Scale for Assessment of Positive Symptoms (SAPS) and the Scale for Assessment of Negative Symptoms, respectively [27]. All patients were taking atypical antipsychotic drugs (risperidone, olanzapine, or clozapine) at the time of testing.

Twelve healthy and medication-free controls (5 females and 7 males) were recruited from the same local community. They had no DSM-IV Axis I diagnosis based on the Structured Clinical Interview for DSM IV (SCID) [28]. Exclusion criteria were history of schizophrenia in themselves or in their families, head injury, neurological disorders, substance use within the past 6 months, and IQ<85. Control participants were also screened before the experiment to rule out elevated schizotypy using the Schizotypal Personality Questionnaire (SPQ) [29]; none of those volunteers had to be rejected on those grounds. Mean (SD) SPQ score was 13.3 (7.2).

All participants had normal or corrected-to-normal visual acuity, and they wore their refractive correction during testing. There were no statistically significant group differences in age, IQ, handedness, or education level. Demographic information is summarized in Table 1.

**Table 1:** The demographic data.

	Control subjects (n = 12)	Schizophrenia subjects (n = 15)	*p*
Age	34.0 (7.8)^A^	40.6 (9.4)	0.053
Sex (M/F)	7/5	8/7	0.79
Education (years)	15.9 (2.1)	14.4 (1.7)	0.063
WASI IQ Score	104.4 (14.1)	95.9 (17.1)	0.19
BPRS	n/a^B^	14.28 (10.13)	
SAPS	n/a	14.21 (11.66)	
SANS	n/a	19.85 (16.63)	
SPQ	14.5 (7.06)	n/a	
Handedness (L/R/Bi) & (Edinburgh score)	2/10/0 60.8 (61.0)	1/13/1 65.7 (41.4)	0.8
Illness duration (years)	n/a	14.7 (8.9)	
Medication (CPZ equivalent, mg/day)	n/a	268.04(107.93)	

A Mean (standard deviation).

B Not applicable.

#### Stimuli

**Figure 1:**
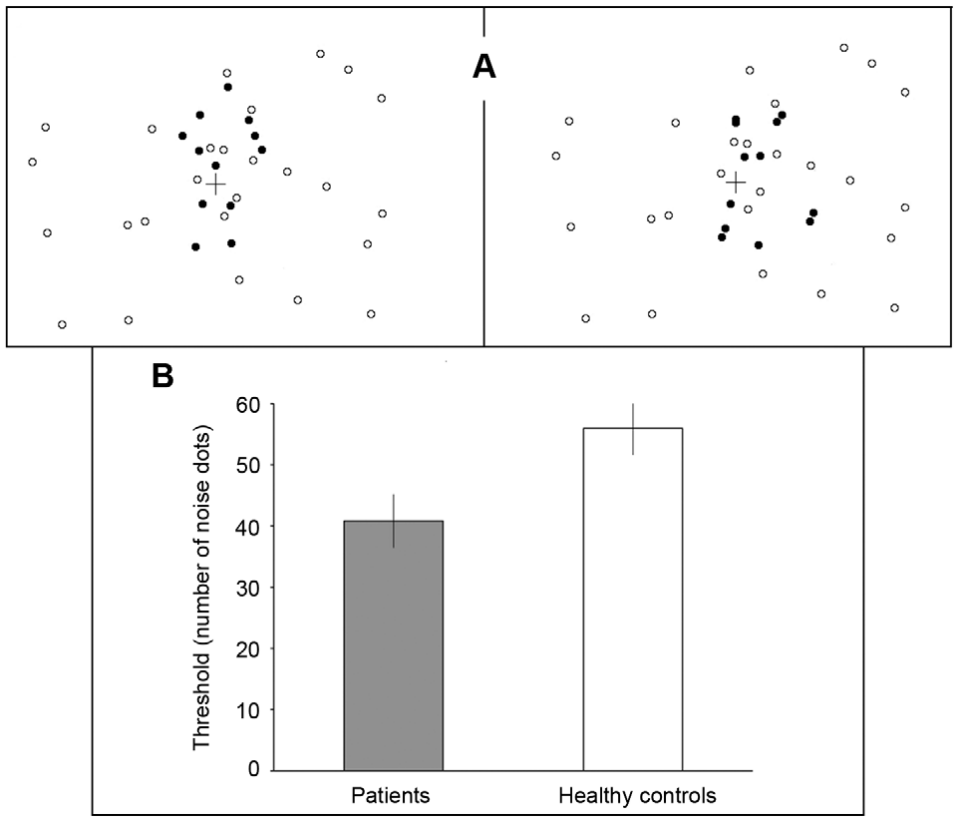
Experiment 1: Detection of biological motion in noise. A. Each trial consisted of two successive 1 sec presentations of PL animations (separated by a 0.5 sec blank period), with one interval containing biological motion in noise and the other interval containing scrambled motion in noise; the scrambled motion on each trial was always derived from the biological motion presented on that trial. The left panel shows one frame depicting biological motion in the first interval (black dots indicate biological motion) and the right panel a frame of scrambled motion in the second interval. In the actual experiment all dots appeared as black against a white background. Noise dots had the same local motion trajectories as those of the biological or scrambled motion on that trial. The set of biological motion sequences totaled 24 distinct activities: 5 walking (stairway walking, climbing, crossing a small object, and 2 plain walking with different viewing angle), 4 jumping (standing jump, leaping, rope-jumping, and high-jumping), 4 kicking (toward front, side, and 2 soccer kicking), 2 running (plain and turning around), 6 throwing (3 overhead and 3 under-throwing), and 3 crouching. B: Results of the biological motion detection task. Mean detection thresholds for the two groups are shown, together with error bars indicating ±1 standard error of the mean (SE).

Animations consisting of black dots presented against a white background were presented on a CRT monitor (120 Hz, TOTOKU Calix CDT2141A, Japan) controlled by a PowerMac G5 computer (Apple Inc, Cupertino, CA) running Matlab© (Mathworks Inc. Natick, MA) and the Psychophysics Toolbox [30], [31]. The experiment was conducted in a dark room illuminated by the screen only, with a 64 cm viewing distance maintained by stabilizing the observer's head using a chin/head rest. Biological motion animations consisted of 12 dots denoting the locations of the head, torso and joints of a human body engaged in one of 24 distinct activities. Scrambled motion sequences of each of those 24 activities were created by randomizing the spatial locations of the dots in the first frame of a sequence. The difficulty in discriminating biological from scrambled animations was manipulated by presenting each animation within a field of noise dots (see Figure 1A). The motion trajectories of the noise dots corresponded to those of biological or scrambled motion sequences on the same trial. This form of masking is particularly effective in degrading perception of biological motion [24], [32].

#### Task

On each trial of this 2AFC task, the participant maintained fixation on a small cross located at the center of the display monitor while viewing two successive, 1 sec sequences (separated by 0.5 sec blank interval) defining a trial. One interval contained the dot sequence defining a biological activity in noise and the other contained a corresponding scrambled sequence within the same level of noise. Following the two successive presentations constituting a trial, the participant pressed one of two keys on a computer keyboard to indicate which of the two intervals contained the biological sequence, guessing if necessary. Auditory feedback was provided following incorrect responses. The number of noise dots presented on a given trial was governed by a two-up/one-down staircase procedure that converges onto the noise level producing approximately 71% correct performance. The staircase was terminated after 16 reversals, and the threshold was estimated as the average number of noise dots over the last six reversals. A sequence of trials began with 20 noise dots, and the noise levels were incremented and decremented in steps of 6 noise dots per change for the first 12 reversals in the staircase and in steps of 3 noise dots per change after that.

The size of each dot was 5-arc min, and the average dot speed within a sequence was 4°/sec. The entire array of dots, noise dots included, appeared within a virtual square region approximately 11° on a side, and the cluster of 12 dots defining biological or scrambled motion fell within a square region subtending approximately 7° on side centered on the fixation mark. The exact spatial location of the biological figure and the corresponding scrambled figure was varied from trial to trial by 1.4 deg visual angle around the center of the noise field; this maneuver made it impossible for participants to monitor just a small subset of dots to judge which interval contained the biological sequence.

### Results

Mean (SE) noise levels (estimated threshold) are shown in Figure 1B, and those values were were 40.83 (4.39) and 55.96 (4.36) for the schizophrenia group and for the control group, respectively. This difference is statistically significant (t(26) = 2.43, p<0.03). Response times were not recorded on a trial-by-trial basis, but the total elapsed time was not significantly different between groups (t = 1.22, p = 0.23). It is unlikely, therefore, that the performance differences are attributable to differences in the length of time taken to arrive at a decision following each trial. We also analyzed the trial-by-trial performance of each participant, to learn more about the pattern of correct and error responses. The mean (SE) number of trials for all staircases was 62.31 (2.31) in the control group and 56.33 (2.11) in the schizophrenia group; this difference is not statistically significant (t = 1.91, p = 0.067). A participant had to be correct on the first 2 trials of the session for the staircase to proceed to the next level of noise. Eight out of 12 control participants responded correctly on the first two trials, compared to only 5 out of the 15 patients. This difference was significant (Pearson χ2 = 4.41, p = 0.036). Thus individuals in the schizophrenia group not only had more difficulty discriminating PL sequences in noise, they also performed worse on the easier trials. These early errors are not surprising, since our earlier study [13] found that patients tended to confuse scrambled and biological motion even in the absence of noise.

There were no significant correlations between performance on the task and 1) symptom severity (BPRS: *r* = 0.17, *p* = 0.57; SAPS: *r* = 0.16, *p* = 0.58; *r* = −0.14, *p* = 0.63) 2) other demographic variables (age: r = −0.22, p = 0.43; education: r = 0.24, p = 0.39; IQ: r = −0.02, p = 0.95; illness duration: r = −0.23; p = 0.46; Edinburgh: r = 0.10, p = 0.72) and 3) medication (r = −0.09; p = 0.73).

These results indicate that schizophrenia patients experienced greater difficulty distinguishing biological from non-biological motion sequences when those sequences appeared within an array of distracting noise dots, compared with healthy controls. Specifically, the level of noise had to be approximately 30% lower for schizophrenia patients to perform at the same level of accuracy as controls. One could argue that this deficit is attributable to a more general impairment involving perceptual organization and figure/ground segmentation. Indeed, schizophrenia patients exhibit impaired performance on some tasks requiring integration of spatially distributed visual features [33], [34], although they perform equivalently to healthy individuals on other perceptual organization tasks [13], [35], [36]. Successful discrimination performance in the present experiment requires spatiotemporal integration of the PL motion tokens signifying a given human activity, and that integration process also depends on successful figure (PL motion)/ground (noise) segregation.

To target the process of spatiotemporal integration that is not confounded by figure/ground segregation, we employed an entirely different task without noise elements in the second psychophysical experiment: a more subtle, challenging task that depends crucially on the ability to judge spatiotemporal coherence in PL animations that are uncontaminated by extraneous noise dots. Because it was impossible to create these kinematically perturbed animations adaptively in real time, we had to administer this task as a method of constant stimuli, not an adaptive staircase procedure.

## Experiment 2: Perceptual discrimination of perturbations in biological motion sequences

In Experiment 2, we compared how well schizophrenia patients performed, relative to healthy controls, on a task involving discrimination of pairs of PL sequences that differed in their degrees of spatial perturbation of the dots defining a biological activity. With these kinds of sequences, small amounts of perturbation preserve the gross impression of biological motion only up to some degree of perturbation, after which the sequences look incoherent. On each trial, two differently perturbed PL sequences generated from the same normal biological motion were presented simultaneously and the participants were asked to indicate which one of the two motion sequences looked more normal.

### Materials and Methods

#### Participants

Participants included all individuals in Experiment 1 along with 6 new participants (2 patients and 4 controls). The two groups were matched demographically, and those demographic data are shown in Table 2.

**Table 2:** The demographic data.

	Control subjects (n = 16)	Schizophrenia subjects (n = 17)	*p*
Age	35.6 (2.47)^A^	39.6 (9.3)	0.22
Sex (M/F)	8/8	10/7	0.61
Education (years)	15.5 (2.23)	14.4 (1.7)	0.12
IQ	103.8 (12.9)	101.8 (22.7)	0.76
BPRS	n/a^B^	15.1 (9.8)	
SAPS	n/a	14.9 (11.1)	
SANS	n/a	21.1 (15.9)	
SPQ	13.3 (7.2)	n/a	
Handedness (L/R/Bi) (Edinburgh score)	2/14/0 64.7 (57.6)	1/15/1 66.5 (38.8)	0.92
Illness duration (years)	n/a	15.1 (8.5)	
CPZ equivalent (mg/day)	n/a	246.97 (119.3)	

A Mean (standard deviation).

B Not applicable.

#### Stimuli

**Figure 2:**
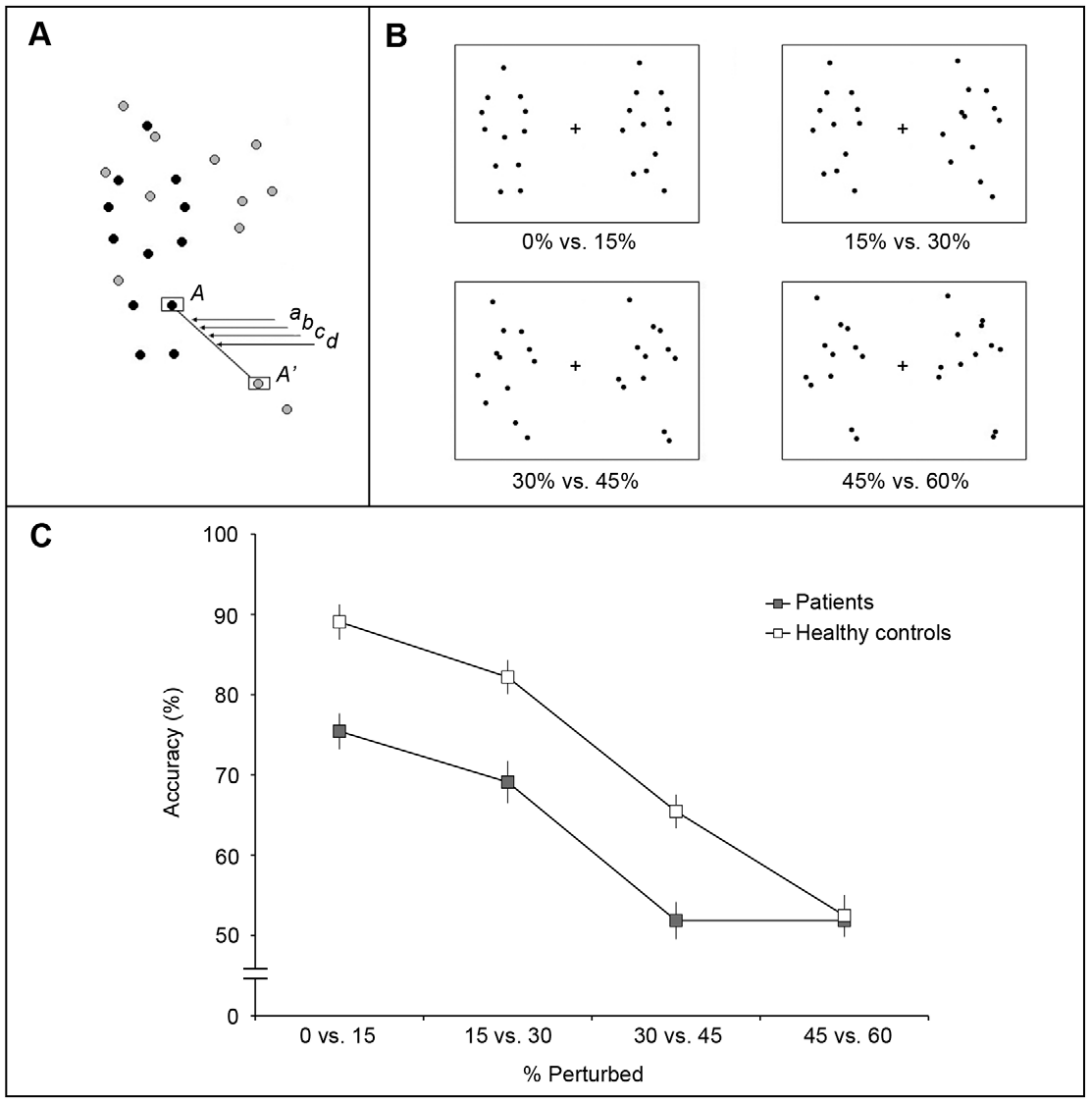
Experiment 2: Discrimination of perturbation of biological motion. A: A series of parametrically perturbed motion sequences was created from 10 different PL animations each depicting a different human activity. These ten different PL animations comprised 2 portraying jumping (standing jump, rope-jumping), 3 kicking (toward front, toward side, and soccer kicking), 3 throwing (tossing, bowling, overhead throwing), 1 crouching for high jump, and 1 backward walking. In this example, black dots indicate the dots forming a single frame of a normal biological PL sequence and gray dots illustrate the corresponding frame of spatially scrambled version of this sequence. For example, dot A' indicates a new location of dot A when the motion is 100% spatially scrambled. The position denoted as (a) corresponds to an intermediate position that divides the distance between A and A' in the ratio of 15∶85. Therefore, when the position ‘(a)’s are taken from all the other pairs of biological-scrambled dots, a sequence containing 15% perturbed biological motion is generated. In the same way, (b),(c), and (d) represent the dot positions of 30%, 45%, and 60% perturbed motion. B: Single frame exemplars of the four discrimination conditions. Over trials, these pairs of animations portraying differing degrees of perturbation were presented in random order, and following each trial the participant indicated which one (left or right) was closer to unperturbed human motion. The % values below each figure refer to the percent of spatial perturbation. C: Performance (accuracy of discrimination) on the task in the schizophrenia group (filled symbols) and the healthy control group (open symbols). Error bars indicate ±1 standard error of the mean (SE). Chance performance on this 2AFC task corresponds to 50% correct.

A series of parametrically perturbed motion sequences was created from 10 different PL animations (listed in the caption for Figure 2). The graded degrees of perturbation were produced in the following way (see Figure 2A). The starting frame of a given sequence (black dots in Figure 2A) was used to create a corresponding 100% scrambled animation frame in which the initial positions of each dot were spatially randomized within the confines of a virtual display window (gray dots). Next, varying degrees of perturbation from a normal sequence were created by locating each and every dot of an animation sequence a given distance between its normal and scrambled location; animations were generated for each of four values of perturbation ranging from 15% to 60% in steps of 15% (these values were selected based on pilot work). For each of the 10 biological activities we created exemplars of each of the 4 degrees of perturbation, and these exemplars were combined to create the pairs (Figure 2B).

#### Task

Participants were tested using a two-alternative, spatial forced-choice procedure. On each trial, two PL sequences were presented simultaneously for 1 sec, to the left and right of a central fixation mark. Each pair always comprised the same biological activity but the two sequences always differed by 15% in degree of perturbation. Thus on each trial, the participant saw pairs comprising 1 of 4 possible conditions: 0% vs. 15%, 15% vs. 30%, 30% vs. 45%, and 45% vs. 60%. Following each presentation, the participant indicated which motion sequence looked more normal by button press, guessing if necessary. Forty test trials were devoted to each pair of perturbation differences, with the order of trials randomized. Prior to formal testing, each participant viewed multiple examples of the various degrees of perturbation as well as examples of all of the normal biological motion. The two motion sequences presented on each trial fell within a rectangular region subtending approximately 9° (width) and 6° (height). During the presentation of the PL pairs, the participant was allowed to successively fixate the two sequences if desired.

### Results

Mean (SE) accuracy levels for each perturbation condition are shown in Figure 2C, and here it can be seen that even with small degrees of perturbation participants in both groups made errors. A repeated measures ANOVA revealed a significant main effect of perturbation (F(3,93) = 99.93, p<0.001), confirming that all participants had increased difficulty discriminating pairs of animation containing greater degrees of perturbation. A significant main effect of diagnosis was also confirmed by ANOVA (F(1,31) = 28.99, p<0.001): schizophrenia patients were less accurate in discriminating two differently perturbed motion sequences compared to healthy controls. The interaction between diagnosis and perturbation condition was also statistically significant (F(3,93) = 5.17, p<0.01), and this is obvious from the graph: healthy controls showed an approximately linear decrease in discrimination accuracy that fell to the chance level only for the pair of 45% vs. 60%, whereas schizophrenia patients fell to chance for the 30% vs. 45% stimulus pair. We also looked at each individual's performance at each of the four perturbation conditions, to calculate how many observers in the two groups performed above chance as defined by binomial distribution (i.e. 26/40 or greater % correct). Results shown in Table 3 point to the same conclusion: patients found this task generally more difficult than controls except at the highest degree of perturbation where nearly all individuals found the task to be impossible.

**Table 3:** The number of subject who performed above chance accuracy of binomial distribution in each perturbation level.

	0% vs. 15%	15% vs. 30%	30% vs. 45%	45% vs. 60%
Patients (n = 17)	15 (75.44)*	13 (69.12)	3 (51.88)	2 (51.88)
Controls (n = 16)	16 (89.06)	16 (82.19)	9 (65.47)	1 (52.5)

*Mean accuracy (% correct).

**Figure 3:**
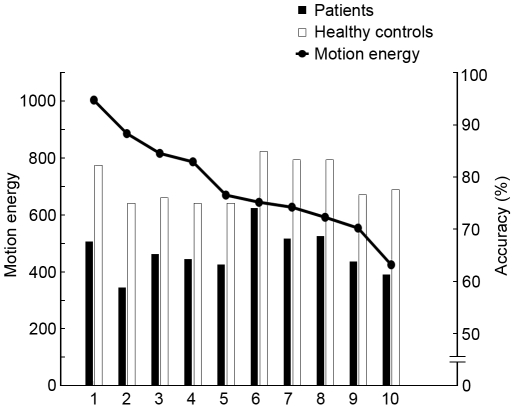
Experiment 2: Performance for each distinct activity. Each activity animation was quantified in terms of the total motion energy in that animation (defined by the total excursion of dots over space and time during the 1 sec presentation). The motion energy of each animation is speicified by the y-axis on the left-hand side of the graph, and the animations are ordered from most to least motion energy along the x-axis. (1 = standing jump; 2 =  kicking side; 3 = crouching jump; 4 = soccer kicking; 5 = kicking front; 6 = backward walking; 7 = bowling; 8 = rope jumping; 9 = tossing; 10 = overhead throwing). The histogram bars show average discrimination performance associated with each activity pooled across all perturbation pairings except the most extreme perturbations where performance on the task was impossible. Filled bars are for schizophrenia patients and open bars for healthy controls. There is no correlation between performance and total motion energy.

In this experiment each trial involved presentation of a pair of PL animations portraying the same activity at two different levels of perturbation, and those PL animations could be any one of ten different activities. Are the group differences in performance on this task dependent on the particular activity being portrayed? To answer that question we computed for each observer the percent-correct performance for each of the ten animations separately, pooling over the different degrees of perturbation (except for the pair of sequences where performance was at chance for both groups). The results of that analysis, shown in Figure 3, confirm that degree of perturbation was more difficult to distinguish for some PL animations compared to others, but the pattern of results was the same for healthy controls and patients, with the correlation between groups being highly significant (r = 0.85, p = 0.002). The variations in task difficulty associated with the different animations, in turn, led us to wonder whether those variations were related to the amount of body and limb motion associated with the different activities. To estimate the overall amount of motion in each of the ten motion exemplars, we derived an index of motion energy for each exemplar defined as the total angular deviation produced by each dot of a given unscrambled animation during one cycle of the activity being portrayed. Those index values, also shown in Figure 3 for each motion type, confirm what is obvious from visual inspection of the animations, namely that some entail larger body and limb motions than others. But the correlation between these index values for each motion type and the associated percent-correct performance for each type indicates that the two factors are unrelated, both for healthy participants (r = −0.11, p = 0.76) and for patients (r = −0.03, p = 0.92).

We examined whether patient performance was related to symptom severity. Here we found that performance (mean accuracy of the scrambling conditions excluding 45% vs. 60% condition) and symptom severity were not significantly correlated (BPRS: r = 0.31, p = 0.91, SAPS: r = −0.13, p = 0.96, SANS: r = 0.37, p = 0.15). Other demographic variables were also uncorrelated with performance (age: r = 0.198, p = 0.45, education: r = 0.24, p = 0.36, IQ: r = 0.39, p = 0.13, handedness: r = 0.17, p = 0.51, illness duration: r = 0.09, p = 0.74, medication: r = −0.12, p = 0.63).

One possible cause of the observed deficit of biological motion perception is that patients may be generally less sensitive to the spatio-temporal coherence defining normal body movements. According to this view, schizophrenia patients might need more salient spatio-temporal coherence to gain an impression of biological motion; sequences with relatively large degrees of perturbation appear equally disordered and therefore indiscriminable. Alternatively, it could be that perturbed sequences strongly resemble coherent biological motion to the patients, to the extent that both sequences of a pair look normal and hence indistinguishable. The higher false alarm rates exhibited by schizophrenia patients in our previous study and in the behavioral task of Experiment 3 are certainly consistent with this second alternative. In the Discussion we consider this second alternative in greater detail.

The results from Experiments 1 and 2 set the stage for examining possible neural concomitants of the impaired ability of schizophrenia patients to discriminate biological motion sequences, a heretofore-unexamined question.

## Experiment 3: An event-related fMRI study of biological motion perception

From human brain imaging studies, there is a growing body of evidence for the existence of a network of dorsal and ventral stream cortical areas involved in the analysis of kinematic information defining human action [1]. One key component in that network is found in the posterior portion of the superior temporal sulcus (STSp). Within this area, neural responses are stronger when one views motion of a human figure or human-like robots [37], PL biological sequences [16], [17], [38], [39] or biological motion in noise [23]. In contrast, STSp is not strongly activated by scrambled PL sequences, by isolated pendular motions or by mechanical motions lacking purposeful meaning [37]. The behavioral results from Experiments 1 and 2 naturally lead to the following question: Are patterns of brain activation in schizophrenia different from those in healthy individuals?

In Experiment 3, we used event-related fMRI to measure the BOLD activity levels associated with viewing biological motion sequences while participants–schizophrenia patients and normal controls–performed a biological motion discrimination task that allowed us to analyze separately brain activations measured on correct trials and error trials. For brain scanning we targeted STSp as well as motion-sensitive area MT, a neighboring visual area that presumably implicated in deficient motion perception in schizophrenia [9], [10], [40], [41].

### Materials and Methods

#### Participants

Ten outpatients with schizophrenia (4 females and 6 males) and ten healthy controls (5 females and 5 males) participated in the experiment. Summary of demographic information is shown in Table 4.

**Table 4:** The demographic data.

	Controls (n = 10)	Patients (n = 10)^C^	p
Age	38.7 (7.2)^A^	41.7 (9.42)	0.43
Sex (M/F)	5/5	6/4	0.65
Education (years)	15.7 (2.7)	14.3 (2.45)	0.24
IQ	101.9 (11.8)	100.3 (27.89)	0.87
BPRS		14.9 (6.6)	
SAPS		19.6 (15.24)	
SANS		28.8 (14.9)	
SPQ^B^	14.3 (9.0)		
Hand (L/R/Bi) (Edinburgh score)	0/10/0 92.5 (10.1)	2/7/1 53.0 (59.6)	0.053

A Mean(Standard deviation).

B Schizotypal Personality Questionnaire.

C Two patients out of twelve were excluded from analyses because of lack of behavioral response on the task during fMRI scans.

#### Stimuli

The same series of 24 distinct biological activities and corresponding spatially scrambled motions used in Experiment 1 were presented at the center of the screen (note that, unlike in Experiment 1, these sequences were not embedded in noise). Each PL animation fell within a virtual rectangular region subtending approximately 3.0×6.0° visual angle. Animations consisted of 20 frames displayed within a 1 sec period (50 msec/frame). In addition to the normal biological motion and completely scrambled motion sequences, a series of partially (37%) perturbed motion sequences was also used. The spatial perturbation value of 37% was selected based on the result from Experiment 2: schizophrenia patients performed at chance level when required to discriminate 30% vs. 45% perturbed biological motion, whereas controls exhibited above chance accuracy (65.47%) for this pair of perturbations (Figure 2C). Since only one sequence was displayed per trial, we decided to use the degree of scrambling falling midway between 30% and 45% perturbation.

#### Functional localization of STSp and MT

**Figure 4:**
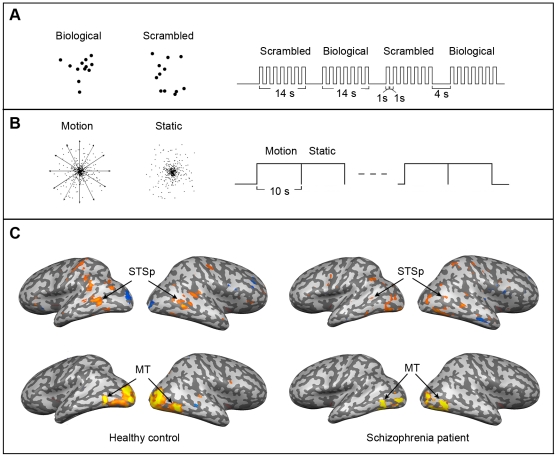
Experiment 3: Localization of regions of interest. A. Stimuli and procedures used to localize area STSp. Shown on the left are examples of PL biological motion and scrambled motion, and on the right is shown schematically the block-designed runs for STSp localization. B. Stimuli and procedures used to localize area MT. Dots moving radially inward and outward and static dots were presented in block-designed runs for MT localization. C. Inflated whole-brain images (both hemispheres for one patient and for one healthy control) showing regions of interest identified using the localizers described above.

Preceding the event-related fMRI scans, we used conventional displays and subtraction techniques to localize areas STSp and MT. STSp was identified by comparing the BOLD signals associated with viewing biological and scrambled motion animations in a block-designed procedure (Figure 4A). Each participant viewed alternating biological and scrambled motion blocks (7 blocks each lasting 14 sec). In each block, seven 1 sec animations were displayed with an inter-stimulus interval of 1 sec. To maintain the observers' attention, each block required performance of a 1-back task in which observers were required to press a button whenever the current motion sequence was identical to the one appearing in the immediately preceding 1-sec presentation; the probability of a repeated sequence was 0.50. The scan lasted 316 sec, with the initial 8 sec (4 volumes, 1TR = 2 sec) being discarded from analyses to allow for MR saturation.

To localize MT, the participants viewed fourteen motion blocks interleaved with fourteen static dot blocks and pressed a button at every point of block switching (Figure 4B). The scan lasted 300 sec. The motion sequence consisted of 380 dots (black against a light gray background) that moved inward and outward from the center of the display. The entire array of the dots fell within a virtual circular region subtending 13° visual angle. The static dot field had the same number of dots, but consisted of only 1 frame. Each dot was approximately 6-arc min in size.

#### Event-related fMRI task

**Figure 5:**
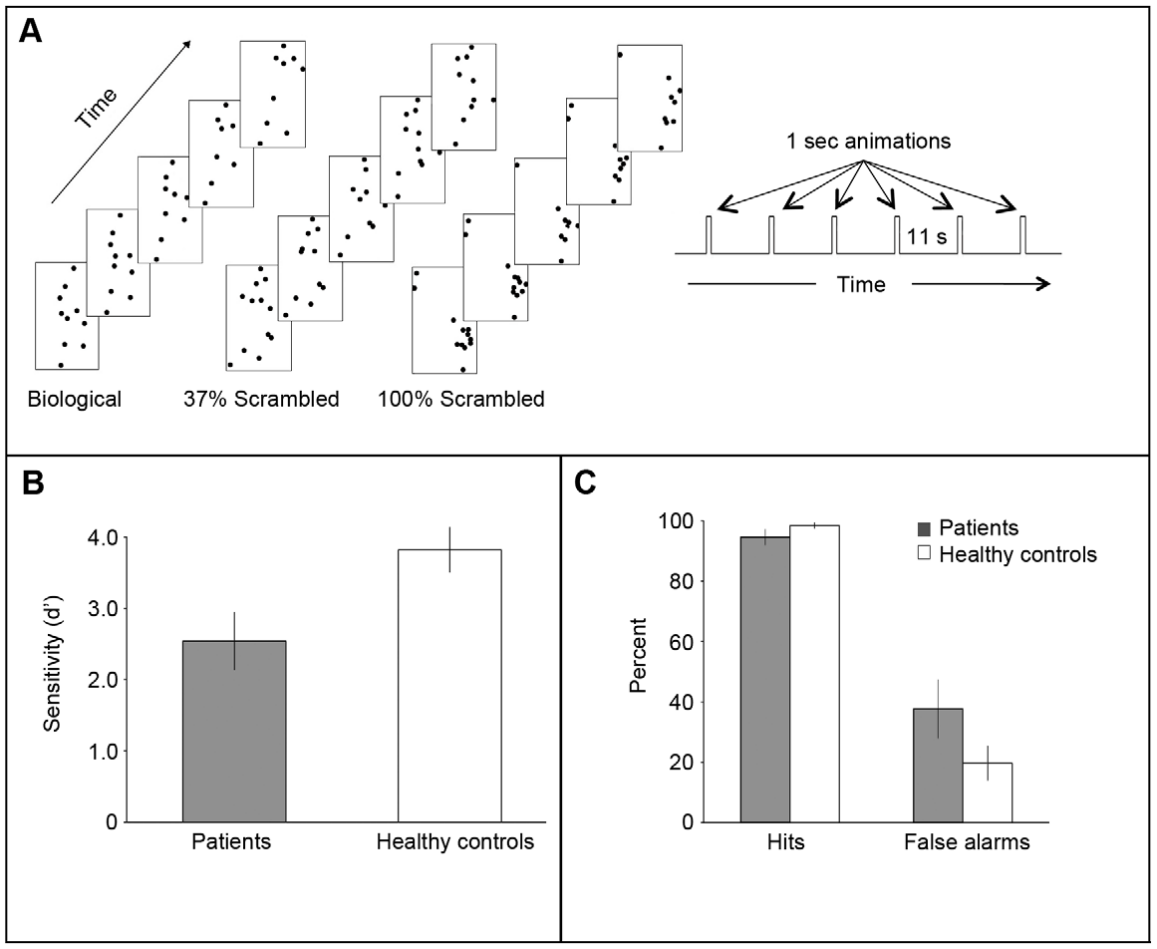
Experiment 3: Event-related portion of brain imaging study. A. The schematics of successive animation frames shown on the left are examples of the three categories of PL animations presented in an event-related fMRI design shown on the right. B. Mean(SE) *d*' on the biological motion task performed during the event-related functional scan. C. Hit and false alarm rates associated with the d' values shown in panel B.

The event-related design for the biological motion task comprised nine runs each containing 24 trials consisting of eight biological, scrambled, and 37% scrambled motion sequences in random order (Figure 5A). We elected to use a constant inter-stimulus interval of 11 sec, to insure that the hemodynamic response associated with a given stimulus presentation had returned to baseline before the next presentation [42]. The participant was always aware of the timing of the next, forthcoming stimulus because the fixation cross changed size 2 sec before that event. Immediately following each stimulus presentation, participants judged whether the given motion depicted a human activity or not by pressing one of two pre-assigned buttons of the hand-puck being worn in the scanner. The total number of trials was 216, and participants were allowed to rest between runs if they so requested.

#### Image acquisition

All brain images were collected on a Philips Intera Achieva 3T MRI scanner located at the Vanderbilt University Medical Center, Nashville, TN. High-resolution T1 anatomical images were collected for each participant (170 slices, 1.0×1.0×1.0 mm). Functional images (single-shot EPI, TR = 2000 ms, TE = 25 ms, flip angle = 90°, matrix = 128×128, FOV = 240×240 mm) were acquired over the whole brain, parallel to AC-PC line (25 slices, 1.875×1.875 mm in plane, 4.5 mm thick with 0.45 mm gap). Visual stimuli were presented using a DLP projector connected to a Macintosh G4 computer (Apple Inc., Cupertino, USA). The projector's image was back-projected onto a screen located at the observer's feet and viewed through a periscope mirror attached to the head coil.

#### Image analysis

Imaging data were preprocessed and analyzed using Brain Voyager QX 1.10 (Brain Innovations, Maastricht, The Netherlands). The anatomical volumes were transformed into stereotaxic space [43], and functional volumes for each participant were aligned to these transformed anatomical volumes. Functional volumes were also preprocessed following procedures including realignment, three-dimensional motion correction, linear de-trending, high-pass temporal frequency filtering, and spatial smoothing with a 4 mm FWHM spatial filter.

To localize regions of interest (ROIs), the general linear model (GLM) was applied to the time-series of task-related functional volumes. ROIs for each individual were then defined as contiguous voxels within the anatomical region of cortex corresponding to the caudal portion of the superior temporal sulcus that were significantly activated by biological motion relative to scrambled motion at a false discovery rate (FDR) of q<0.05, or p-value (uncorrected) lower than 0.003 (if STSp is not successfully localized at the given FDR). The same analysis was applied to voxels in the general anatomical region of the human MT+ complex, this time contrasting activations to optic flow and static dots. Among observers the numbers of voxels identified by these methods ranged from 15–174 in STSp and 38−263 in MT+.

To analyze the functional imaging data, the design matrix (reference time course) was defined to include 4 predictors based on each individual's behavioral response: (1) activation associated with hits (“biological” response to biological motion), (2) activation associated with correct rejection (“scrambled” response to scrambled motion), (3) activation associated with false alarms (“biological” response to scrambled motion), and (4) overall activation to 37% scrambled motion. While there is no objectively correct answer for trials involving 37% scrambled motion, we initially intended to analyze the fMRI results for those trials based on observers' perceptual judgement (“biological” vs. “scrambled”); as reported in the Results, however, there were too few “biological” responses to make that possible. Miss trials (“scrambled” responses to biological motion) also had to be excluded from fMRI analyses because of the paucity of these trials. Within the defined ROIs, the voxels coupled with the event-related trials were averaged to create a single time series for each condition (predictors) in each individual. MR signal levels coupled with each condition were averaged to create an estimate of BOLD activity through the process of event-related averaging in Brain Voyager QX. Percent change in BOLD signal associated with each condition was defined as difference between baseline (activation at the stimulus onset) and the peak activity following stimulus onset. That peak was identified from the actual BOLD signal values plotted over time, not estimated from fitted hemodynamic response functions.

### Results

#### Behavioral results

On the biological motion and scrambled motion trials, schizophrenia patients had significantly lower discrimination sensitivity (*d*') compared to healthy controls (Figure 5B), consistent with our earlier study [13]. Mean (SE) *d*' was 2.54 (0.4) in patients and 3.82 (0.32) in controls (t(18) = 2.45, p = 0.024). Both groups had high hit-rates (controls: 98.4 (1.08)%, patients: 94.6(3.42)%, p = 0.2). The difference in the incidence of false alarms was large (Cohen's *d = *0.75) but failed to achieve statistical significance (controls: 19.7(5.8)%, patients: 37.7(9.7)%, p = 0.13,) (see Figure 5C). Behavioral results from the 37% scrambled motion trials reveal that patients and controls tended to categorize these animations as scrambled, although the incidence of biological responses was larger in the patient group (19%, on average) compared to the control group (4%, on average). Strictly speaking, these “biological” responses cannot be categorized as incorrect, because sequences with 37% scrambling do look more biological than 100% scrambled sequences. (In informal pilot testing of controls and schizophrenia patients, all participants rated both 30% and 45% sequences as more human-like than 60% scrambled sequences, so 37% is undoubtedly seen as different from scrambled.) Still, the higher incidence of “biological” responses from schizophrenia patients viewing the 37% scrambled sequences certainly comports with results from our earlier study [13] where patients had higher false alarm rates than normal controls. Unfortunately, the number of these kind of trials was insufficient to permit analysis of imaging data on trials where 37% scrambled was judged biological.

The behavioral performance (as indexed by d') of the patients measured while they were in the scanner was not significantly correlated with the severity of their clinical symptoms as indexed by rating scales (BPRS, SAPS, and SANS).

#### Localization of STSp and MT

In nine healthy participants, the STSp was functionally localized by subtracting activation to scrambled motion from activation to biological motion at the threshold of q(FDR)<0.05. In the tenth healthy control participant, STSp was localized by applying p<0.003 (uncorrected) because the area was not clear at q(FDR)<0.05. Among the ten members of the schizophrenia group, STSp was localized in six individuals at the threshold of q(FDR)<0.05. For two other patients, that threshold level had to be adjusted to p<0.003 (uncorrected) to localize STSp. For the remaining two patients, STSp could not be localized even by this more lax criterion. As an alternative, the location of STSp in their brains was estimated by identifying significant BOLD activations during biological motion blocks relative to baseline and then delimiting the activated voxels to just those successfully localized in other eight patients.

Area MT was successfully localized in all participants by contrasting activation to optic flow stimuli with that to a static dot field.

Mean (SD) Talairach coordinates of the two ROIs are shown in Table 5, which is similar to those of a previous study [23]. For reference, Figure 4C shows those ROIs–STSp and MT–in one normal control and one schizophrenic patient.

**Table 5:** Talairach coordinates of the defined ROIs.

Talairach Coordinates							
		Left hemisphere			Right hemisphere		
	ROI	*x*	*y*	*z*	*x*	*y*	*z*
CO	STSp	−51.3 (5.56)	−58.0 (8.72)	10.3 (5.7)	49.5 (7.6)	−53.5 (8.9)	9.6 (5.3)
	MT	−43.2 (4.2)	−69.1 (5.0)	−0.9 (5.0)	44.1 (1.9)	−63.8 (5.6)	0.4 (5.2)
SZ	STSp	−51.1 (7.5)	−55.6 (8.1)	8.6 (5.1)	48.1 (9.1)	−54.6 (10.8)	9.1 (3.9)
	MT	−42.6 (5.42)	−69.8 (6.1)	−1.11 (4.63)	43.0 (3.68)	−64.7 (5.0)	−0.58 (5.6)

Mean coordinates and standard deviations (in parentheses) for the two ROIs (posterior STS and MT) in each group. CO: controls, SZ: schizophrenia patients.

#### Event-related activity in STSp

**Figure 6:**
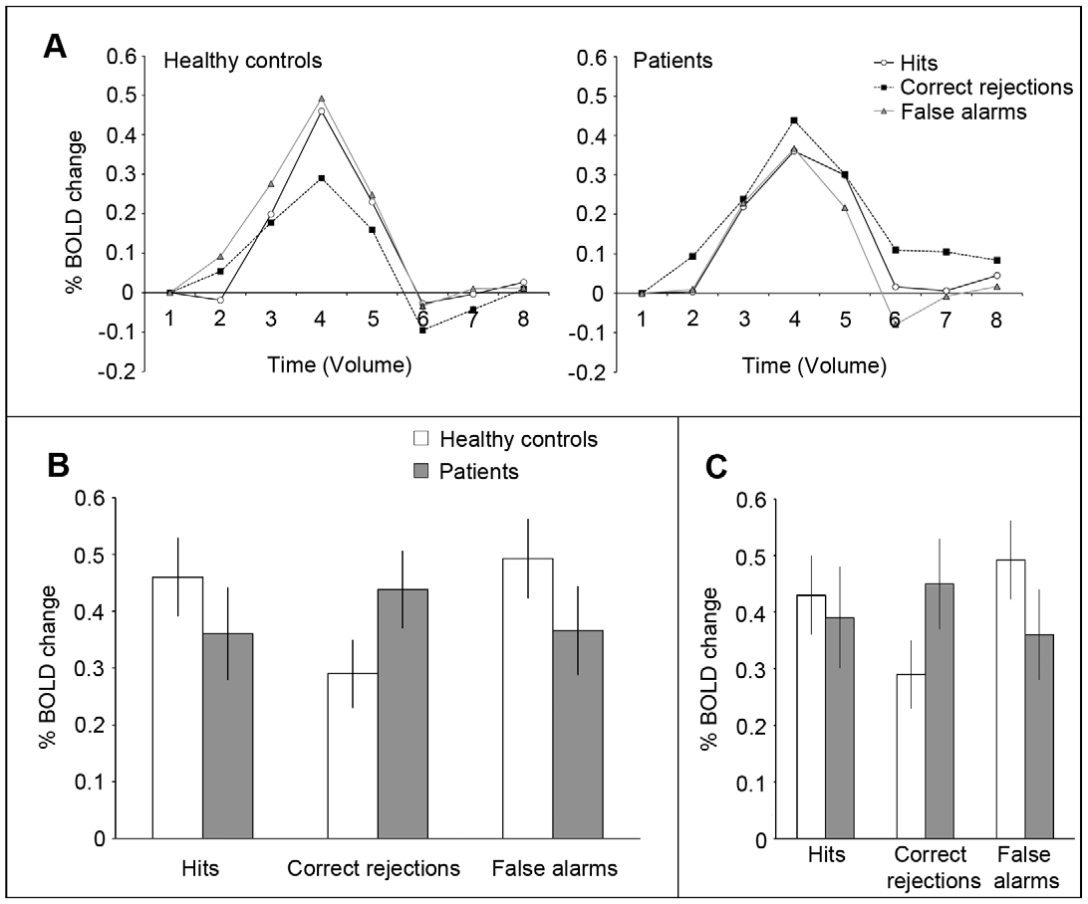
Experiment 3: Brain imaging results from STSp. A: Average time-series associated with each of three signal detection categories (hit, correct rejection, false alarms) in controls (left) and patients (right). Time value 1 denotes stimulus onset (TR = 2 sec). B: Each histogram plots, for patients and healthy controls, the peak BOLD signal levels (1 SE) associated with each of the three signal detection categories. C. Same as panel B, with data removed for the two schizophrenia patients for whom STSp localization was based on anatomy, not differences in activations on the STSp localizer.

Group averaged peak BOLD responses for hit, correct rejection, and false alarm trials are shown in Figure 6A. We have no control, of course, over the number of trials contributing to these three categories, for those categories are defined by the stimulus *and* by the participants' responses. Because hit rates were higher than false alarm rates for both groups, more fMRI BOLD signal estimates comprise the responses associated with hits than with the other two categories, but this is true for both healthy controls and patients. Moreover, a multifactorial repeated measures ANOVA confirmed that overall activations across these three categories did not differ significantly between groups (F(1,30) = 0.031, p = 0.86). The main effect of the signal detection category was not significant, either (F(2,60) = 0.93, p = 0.40), but the interaction between signal detection category and diagnosis was significant (F(2,60) = 4.57, p = 0.014). ANOVA with two selected categories also revealed significant interaction effects (hit vs. correct rejection: F(1,30) = 6.89, p = 0.01; correct rejection vs. false alarm: F(1,30) = 9.63, p = 0.004). These statistical analyses confirm the impressions portrayed by the patterns of results seen in the summary data in Figure 6B.

Summarizing those results for the two groups separately, healthy individuals produced significantly greater STSp activation on hit trials (biological motion perception) than on correct rejection trials (scrambled motion perception) (F(1,16) = 12.05, p = 0.003). Interestingly, STSp activations on false alarm trials were not significantly different from activations on hit trials (F(1,16) = 1.22, p = 0.29), suggesting that people with strong STSp activation on a given trial tend to perceive the animations presented on those trials as biological motion. This correlation between perceptual state and brain activation has been reported for other visual tasks as well [44], and it is a point we return to in the Discussion. On the other hand, schizophrenia patients did not show differential activation for the three signal detection categories: levels of STSp activation within patients were not significantly different across hits, correct rejections and false alarms (F(2,24) = 0.512, p = 0.65). The same conclusion is reached when we perform pair-wise comparisons of hits to false alarms (t(12) = 0.21, p = 0.84), hits to correct rejections ( t(12) = −0.72, p = 0.49) and correct rejections to false alarms (t(12) = 1.107, p = 0.29). This absence of differential activation in patients quite plausibly could contribute to their poor ability to discriminate biological motion from scrambled motion, for within STSp those two categories of animations produce highly similar levels of activity.

It is natural to wonder why STSp in patients showed no differential activation on hit and correct rejection trials event though the two categories of PL animations presented on those trials–biological and scrambled–were used successfully in 8 out of 10 patients to identify STSp on the localizer trials. These differential results, we surmise, are attributable to the fact that block designs typically generate more robust BOLD signals than do event-related designs. This was certainly true for our experiment: average peak activations (biological and scrambled) for STSp in patients averaged 0.59% in the block design but only 0.4% in the event-related design. The tasks, too, were different for the two designs, although it is not obvious why the one-back task (used in the block design) would promote differences between scrambled and biological whereas the categorization (used in the event-related design) would not. We will consider the general question of task performance and event-related imaging results further in the Discussion.

As mentioned earlier, STSp was not successfully localized using conventional statistical methods in two of the ten schizophrenia patients. To be sure their results were not responsible for the lack of BOLD signal differences between scrambled and biological sequences in the schizophrenia group, we reanalyzed the group data with those two individuals' data removed (n = 8). This additional analysis yielded the same pattern of results (Figure 6C): the main effects of diagnosis and of signal detection category were not significant (F(1,28)<0.001, p = 0.99; F(2,56) = 0.67, p = 0.52, respectively) and the interaction effect was significant (F(2,56) = 4.22, p = 0.02). Interaction effects between two signal detection categories (hit vs. correct rejection; correct rejection vs. false alarm) were also significant (F(1,28) = 5.2; p = 0.03; F(1,28) = 9.32, p<0.01, respectively). We are thus confident that the two patients in whom STSp was not conventionally localized were not the sole source of the overall differences between patients and normal controls.

As mentioned before, the paucity of “biological” responses in the 37% scrambled condition precluded statistical analyses of the fMRI results for this condition contingent on the perceptual report. We were able, however, to perform group comparisons of the overall activation levels for this stimulus condition irrespective of response category, and those activations did not differ between the groups (t(30) = 0.93, p = 0.36).

Clinical symptom scores from the schizophrenia patients were not significantly correlated with STSp peak activation in any signal detection category.

#### Event-related activities in MT

**Figure 7:**
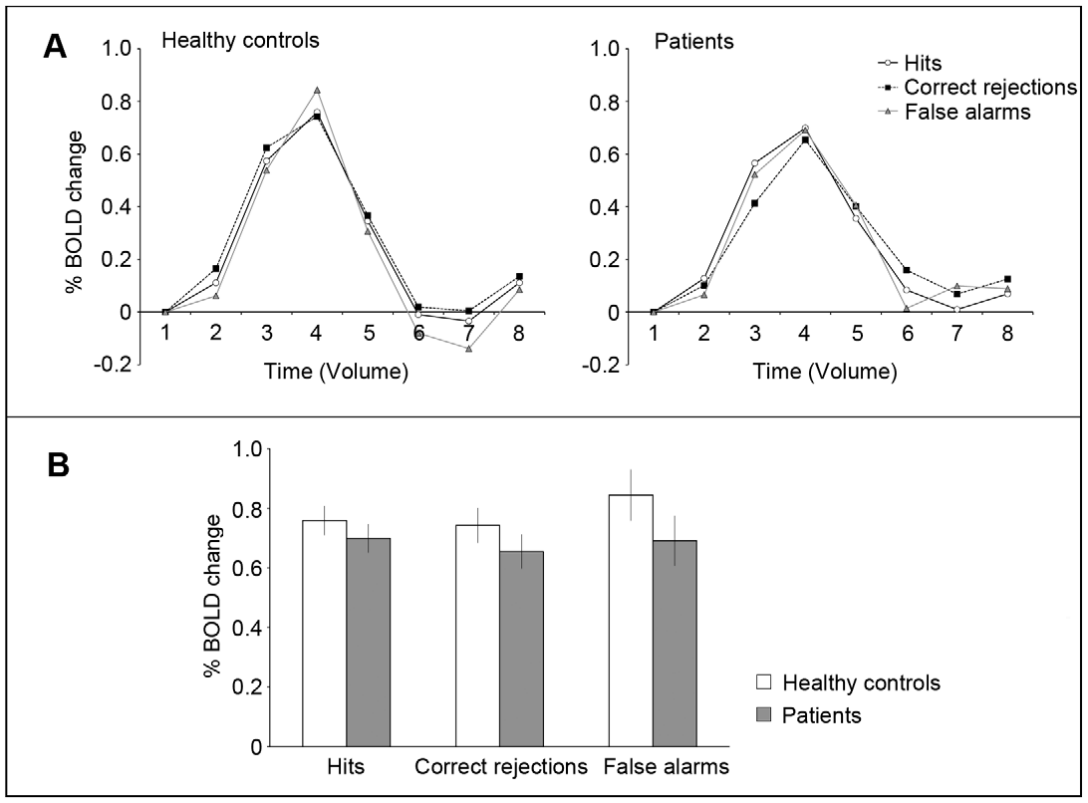
Experiment 3: Brain imaging results from MT. Same format as in Figure 6.

The same analysis procedures were applied to MT activations measured during the biological motion task (see Figure 7). There was no significant group (diagnosis) difference in overall activation (F(1,35) = 2.24, p = 0.14), nor a significant main effect of signal detection category (F(2,70) = 1.43, p = 0.25). Unlike STSp activation, the diagnosis×signal detection category interaction effect was not significant (F(2,70) = 0.18, p = 0.84), indicating that MT activation is not associated with stimulus type or observer's response.

To learn whether MT activity level is related to STSp activation, we computed the correlation between peak activations between the two areas. In healthy controls, there were no significant correlation between STSp and MT activation in any of signal detection categories. In patients, the correlations between STSp activity and MT activity for hit trials and correct rejection trials were not significant. There was a significant correlation on false-alarm trials, but it was restricted to the left STSp and MT (r = −0.88, p = 0.047 in left; r = 0.25, p = 0.51 in right). However, the sample size was small: left STSp was localized in only 5 patients, including one who exhibited extraordinarily strong activation. In general, we see no strong indication that MT activation predicts responses in STSp when results are analyzed contingent on the participants' responses to given categories of stimuli.

## Discussion

The behavioral experiments confirm that schizophrenia patients, compared to healthy controls, experience difficulty distinguishing biological motion from non-biological motion sequences. We have now seen these group differences on three complementary tasks: simple categorization of sequences as biological or scrambled (Reference 13, and the behavioral component of Experiment 3), discriminating biological from scrambled in the presence of distracting noise (Experiment 1) and discriminating biological motion sequences in which the spatio-temporal coherence of the dots defining kinematics is perturbed (Experiment 2). Moreover, we have identified a potential neural correlate of this deficit in the BOLD signals measured from STSp, a brain area known to be involved in perception of biological motion.

Can these results be attributed to the fact that all patients in this study were taking antipsychotic medication at the time of testing and scanning? Past perceptual and cognitive studies with schizophrenia patients have not found significant differences in performance between medicated and non-medicated patients (e.g. [45]), nor have they found a significant correlation between medication and performance [46], which is what we too observed in the present study. We are, therefore, disinclined to believe that medication alone is the sole factor responsible for our patients' performance deficits. What, then are the reasons for these deficits? In the following paragraphs we consider alternative interpretations of these findings.

Starting with the psychophysically measured perceptual deficits in schizophrenia patients, it is reasonable to ask whether they are unique to biological motion or, instead, stem from a more general problem in motion perception. Indeed, earlier work has shown that schizophrenia patients require stronger translational motion signals to discriminate direction of motion in random-dot cinematograms (RDC) containing signal and noise dots [11]. And it is true that our masking study involved detecting biological motion figures embedded in dynamic noise dots, similar to the conventional RDC task. For several reasons, however, we believe that the deficits perceiving biological motion go beyond simply a deficit in perceiving signal dots within noise. First, the discrimination task (Experiment 2) did not involve noise dots, yet deficient performance was observed. The same is true for our earlier task [13] and for the behavioral task employed in our brain imaging study (Experiment 3). Second, the stimulus information supporting detection of weak translational motion within fields of random dots is fundamentally different from the information specifying the hierarchical, pendular motions of dots creating the vivid impression of biological motion. Third, these two disparate forms of motion perception appear to be mediated by distinct neural mechanisms as evidenced by their different integration time constants [47], their dissociation consequent to brain damage [48]–[50], and the different activations they produce during imaging studies in normal people (e.g. [38]). Fourth, neither controls nor patients showed differential MT activation contingent on signal detection categories, and furthermore, we found no meaningful correlations between MT and STSp peak activations in either group. These observations imply that the neural events critical for perception of coherent, translational motion differ, at least in part, from those involved in biological motion perception [1], [51]. Based on four these reasons, we believe the deficits observed on these various tasks involving biological motion sequences are not attributable solely to difficulties perceiving motion in general but, instead, arise from impairments in extracting the kinematics unique to biological motion and effectively isolated using PL animations.

Is it possible that this deficit in perception of biological motion perception in schizophrenia patients is related to a more general problem involving visual grouping of spatially distributed visual elements? We know, for example, that chronic schizophrenic patients are impaired in their ability to recognize objects portrayed in fragmented images in which portions of the contours defining the objects are invisible [52]. This task presumably taps into an ability to fill in missing information using a contour interpolation processes. Perceiving biological activity from PL animations could also be construed as involving interpolation of missing information, in this instance information ordinarily available when viewing whole-body movements and not just the movements of select portions of the body designated by PLs. We have no quarrel with this way of characterizing the nature of the task, and we are intrigued by findings implicating impaired dorsal stream processing as a correlate of deficits in perceiving fragmented objects by schizophrenia patients [53]. After all, STSp is a component of this broad dorsal stream network. We are reluctant to conclude, however, that the neural processes involved in perceiving static fragmented figures are the same as those responsible for perception of dynamic activity portrayed by PL sequences since the latter, but not the former, requires integration of information over time as well as over space. It would be interesting indeed to examine correlations in performance on these rather different tasks in schizophrenia patients and, for that matter, in healthy controls.

In a related vein, it is conceivable that the difficulties experienced by schizophrenia patients when viewing PL animations is somehow related to the well-established abnormalities in temporal integration in these patients [54], [55]. Perhaps in our tasks the 1-sec presentation durations, while adequate for healthy controls, are simply too brief for sufficient visual processing by the patients. We doubt that the presentation duration limited their ability to fixate the displays, for in two of our tasks (Experiments 1 and 3), the PL animations were presented at fixation. In the task involving simultaneous presentation of two animations, saccadic eye movements would be required to achieve successive glances of the stimuli, but existing evidence indicates that simple saccadic eye movements are intact in schizophrenia patients [56]–[58]. But it is possible that limitations in integration of visual information over time contribute to the perceptual deficits documented in our study. Indeed, it is known that temporal summation for perception of biological motion extends beyond one second [47], so an impairment in temporal integration could place patients at a disadvantage relative to healthy controls. It could be informative to assess temporal integration in perception of biological motion in patients, by systematically varying exposure duration.

Turning now to the brain imaging results, in healthy control participants STSp activation was stronger when biological motion was perceived correctly (hits) than when scrambled motion was perceived correctly (correct rejection). This merely confirms what was already known, namely that STSp selectively responds to biological relative to scrambled PL sequences [2], [47], [50], [59]. In contrast, however, schizophrenia patients showed comparable levels of event-related activations in STSp across all signal detection categories, including those where the stimulus involved presentation of scrambled motion. For that matter, we also had more difficulty pinpointing STSp in a couple of our schizophrenia patients using our localization procedure that contrasts biological and scrambled sequences in a simple block design. At present we can only speculate about possible reasons why STSp in these patients produced strong, undifferentiated responses to these different categories of PL animations. It is well known that schizophrenia is characterized by reduced grey matter volume in a variety of brain areas including the superior temporal lobe. Moreover, there is growing evidence that schizophrenia is associated with disordered neural connectivity among brain areas (see review [60]). To the extent that those connections mediate inhibition of during task-related activities [61], we might expect schizophrenia patients to exhibit reduced suppression of activity within brain areas important for registering information kinematics, just as a lack of suppression may underlie their poorer performance on working memory tasks [62].

Given these event-related results from schizophrenia patients, it is natural to wonder what neural information they were using when trying to perform the behavioral tasks we administered to them. After all, their behavioral performance, while reduced relative to normal controls, implies that they could distinguish scrambled from biological sequences at above chance levels. One possibility is that brain areas other than STSp contain neural responses sufficient to signify the nature of the PL animation being viewed [63]. To evaluate that possibility, we looked throughout all brain volumes scanned during event-related brain imaging in our schizophrenia patients, in search of voxels showing reliable signal differences between hit trials (when biological sequences were judged biological) and correct rejection trials (when scrambled sequences were judged scrambled). That whole brain analysis turned up just a few, small clusters of voxels showing significant activation differences. None of those clusters, however, were located within neural structures associated with biological motion activations in normal individuals, leading us to conclude that they were chance differences arising from the multiple comparisons we performed in this analysis.

Alternatively, it is conceivable that on given trials STSp in schizophrenia patients can produce patterns of neural activity that correctly signify the category of animation being viewed (e.g., biological). This possibility is not incompatible with our event-related fMRI results, for those results comprised peak levels of activation averaged over multiple trials for each of the signal detection categories.

One potential clue about the possible involvement of STSp in the performance of schizophrenic patients may come from reconsideration of the false alarm trials and the accompanying brain activations in STSp. Recall that strong STSp activation was observed in healthy individuals on false alarm trials, i.e., error trials on which scrambled motion sequences were seen as biological. Perhaps, then, perceptual errors on false alarm trials-seeing something that is not actually there-are manifestations of neuronal activity ordinarily involved in registering the presence of biological motion. Continuing this line of reasoning, we now know that in schizophrenia patients scrambled sequences produce activations as large as those produced by biological motion sequences, which could well be responsible for their higher false alarm rates on the biological vs. scrambled categorization task (Experiment 3 and Reference 13) and for their general difficulty discriminating biological from scrambled sequences in noise (Experiment 1) or discriminating sequences differing in degree of scrambling (Experiment 2). What we are suggesting, therefore, is that the deficits in biological motion perception in patients are an exaggerated manifestation of the neural events within STSp associated with perceptual errors sometimes made by healthy observers on these same tasks.

Given this possibility, what can we conclude about the origins of the strong STSp activations on false alarm trials? First, it is possible that intrinsic neural noise causes activity levels in STSp to fluctuate spontaneously over time, the consequence being that activity induced by a suboptimal stimulus achieves abnormal levels that mimic activity patterns ordinarily associated with a coherent biological event. This account, however, cannot explain why, in healthy individuals, STSp activity is elevated during visual imagery. Instead, imagery and false alarm-associated activations could result from top-down influences on perception of biological motion, of the sort suggested by earlier work [64]–[68]. For example, efficiency of biological motion processing is strongly influenced by action categories: certain familiar actions (e.g. walking) are generally recognized more quickly and more accurately. This has led to the proposal that high-level vision contains “selective movement filters” [66] or “sprites” [65] that embody models of common actions exhibited by familiar objects including people. Through top-down processes such as attention, these high-level schemas can modulate weak, ambiguous or noisy motion signals and, thereby, bias perception in favor of familiar actions under conditions like those used in our studies (e.g. [68]) One possible candidate for the neural locus of these high-level representations is the inferotemporal sulcus (ITS), an area implicated in object recognition [69], [70], visual imagery [71], and perception of shape configuration [72]. ITS is also known to be responsive to biological motion [73], suggesting that reciprocal connections between STSp and ITS could form at least part of the network involved in top-down influences on perception of biological motion. Regardless of the details of that network, it is clear that such top-down influences may well mediate the strengthened activation within STSp associated with false alarm trials.

To end on a speculative note, our results may fit into the larger discussion about the nature of delusion, a discussion that centers around two themes: faulty perception vs. faulty cognition. The perceptual account explains delusions as the rational explanation of anomalous perception (in other words, the best, correct interpretation of noisy, poor quality sensory data: see [74]). On the other hand, more cognitive account posits that those with abnormal beliefs tend to have cognitive biases that result in faulty hypothesis testing and jumping to conclusions (e.g. [75]–[77]). In fact, however, the two accounts could go hand in hand: poor quality sensory data necessitate increased involvement of cognitive processes to make sense of the world. Indeed there is evidence for complex interaction between cognitive and perceptual information processing that may account for hallucinatory and delusional experiences (e.g. [78]–[80]). Visual information processing is abnormal in schizophrenia (see [81]–[83]) and structural abnormalities have also been observed in the visual cortex [84]. Given that the quality of sensory data in these patients is compromised, they may need to rely more heavily on higher cortical regions (e.g., frontotemporal regions) to make sense of their visual world. Indeed It has been observed that schizophrenic patients give greater weight to top-down expectations on perception than normal controls do [85].

Construed in this context, what we have found in our study is that people with schizophrenia tend to “see” living things in randomness and this subjective experience is correlated with an increased activity in the STSp. This finding is broadly in agreement with past behavioral results suggesting that psychotic or psychosis-prone individuals tend to see meaning where there is none (e.g. [86]), perhaps because they adopt a more lenient criterion for distinguishing perception and imagination owing to abnormal up-regulation of dopamine neurotransmitter [87]. In the case of biological motion perception, these self-generated, false impressions of meaning can have negative social consequences, in that schizophrenia patients may misconstrue the actions or intentions of other people.
